# Electrochemical impedance spectroscopy of human cochleas for modeling cochlear implant electrical stimulus spread

**DOI:** 10.1063/5.0012514

**Published:** 2020-09-01

**Authors:** C. Jiang, S. R. de Rijk, G. G. Malliaras, M. L. Bance

**Affiliations:** 1 Department of Clinical Neurosciences, University of Cambridge, Cambridge CB2 0AH, United Kingdom; 2 Division of Electrical Engineering, Department of Engineering, University of Cambridge, Cambridge CB3 0FA, United Kingdom; 3 Department of ENT, Cambridge University Hospitals Trust, Cambridge CB2 0QQ, United Kingdom

## Abstract

Cochlear implants (CIs) have tremendously helped people with severe to profound hearing loss to gain access to sound and oral–verbal communication. However, the electrical stimulus in the cochlea spreads easily and widely, since the perilymph and endolymph (i.e., intracochlear fluids) are essentially electrolytes, leading to an inability to focus stimulation to discrete portions of the auditory nerve, which blurs the neural signal. Here, we characterize the complex transimpedances of human cadaveric cochleas to investigate how electrical stimulus spread is distributed from 10 Hz to 100 kHz. By using electrochemical impedance spectroscopy (EIS), both the resistive and capacitive elements of human cochleas are measured and modeled with an electrical circuit model, identifying spread-induced and spread-independent impedance components. Based on this electrical circuit model, we implement a Laplace transform to simulate the theoretical shapes of the spread signals. The model is validated by experimentally applying the simulated stimulus as a real stimulus to the cochlea and measuring the shapes of the spread signals, with relative errors of <0.6% from the model. Based on this model, we show the relationship between stimulus pulse duration and electrical stimulus spread. This EIS technique to characterize the transimpedances of human cochleas provides a new way to predict the spread signal under an arbitrary electrical stimulus, thus providing preliminary guidance to the design of CI stimuli for different CI users and coding strategies.

## I. INTRODUCTION

**FIG. 1.**
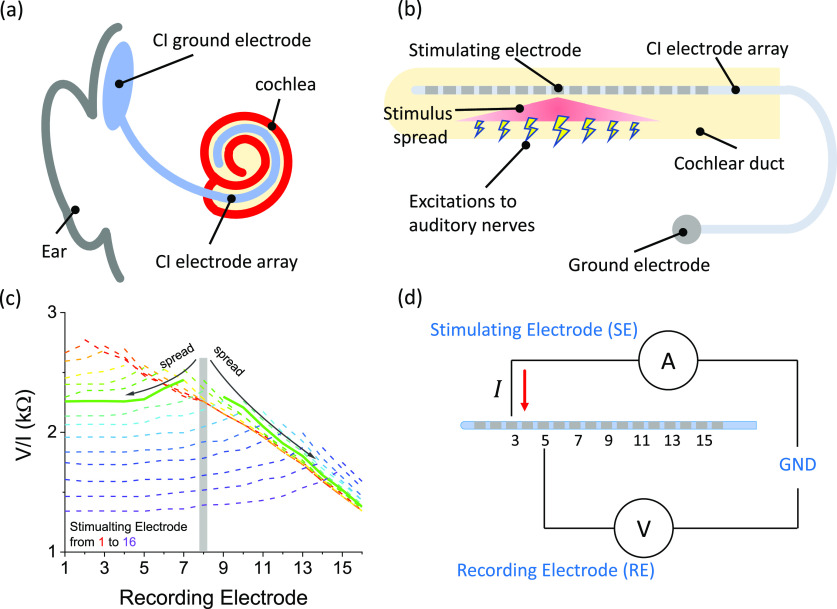
(a) Schematic of a cochlear implantation in a human head. (b) Schematic of the electrical stimulus spread that excites auditory nerves in an unwrapped cochlea. (c) An example of electrical stimulus spread measured with clinical software on a cadaveric cochlea, with the solid curve demonstrating the spread when stimulating electrode 8. (d) The schematic of the setup for cochlear transimpedance spectroscopy and spread-induced voltage measurements.

Cochlear implants (CIs) have helped hundreds of thousands of people who have severe-to-profound hearing loss to improve access to sounds by directly stimulating their cochlear nerves with electrical stimuli.^1^ However, these current pulse stimuli cannot be focused at a narrow region of desired cochlear nerves due to the high electrical conductivity of the fluids inside cochlear ducts (i.e., perilymph and endolymph).^2^ This phenomenon is known as electrical stimulus spread or current spread and results in a severe spectral blurring of the input current stimuli at the neuronal level, as depicted in Figs. 1(a)–1(c).^3,4^ Due to this issue, most CI users experience difficulties in challenging listening conditions (e.g., with background noise or multi-speakers) and have few actual independent information channels, and their musical perception is limited.^5–7^ Clinically, different CI users have different patterns and levels of spread, which result from their individual unique cochlear anatomy and physiology.^8,9^ These properties are difficult to analyze and correlate with stimulus spread, but cochlear impedance is an electrical property that can provide some insight into cochlear stimulus conditions and can be measured clinically.^10^

Cochlear impedances can be measured with the electrode array that is inserted into a cochlea during implant surgery. This electrode array is designed to inject current stimuli into the cochlea and is also integrated with a voltage measurement unit to characterize the impedance of the cochlea. Cochlear impedance measurement can be categorized into two types: contact impedance and transimpedance. The contact impedance is the measured impedance between one intracochlear electrode and the extracochlear ground electrode in the cranial temporal region [Fig. 1(a)], provides useful information to check if the intracochlear electrode is functioning properly, and can reveal open circuits or short circuits in the implant wiring. Contact impedance measurement is used by all of the major CI companies using the stimulus-induced voltage amplitude divided by the input current level, which however does not reveal the true complex impedance.^10^ In addition, contact impedances do not indicate any stimulus spread information, and they are also affected by the electrode/electrolyte interface impedance, therefore not presenting the true cochlear electrical impedances.^11,12^ Transimpedance is the inverse ratio between the delivered current at a stimulating electrode and the measured voltage at another intracochlear electrode, measured in ohms. Since the electrode array of a CI is placed along the cochlea, transimpedance measurement indicates the electrical potential (resulting from the stimulus) built up at the locations away from where a stimulating electrode should focus the stimulus, i.e., the stimulus spread. For commercial CIs, transimpedance measurement is used by several CI companies, using these stimulation-current-induced non-stimulating electrode voltage (SCINSEV) recordings, and is known as the transimpedance matrix (TIM) by Cochlear Corp®, electrical field imaging (EFI) by Advanced Bionics®, and Impedance Field Telemetry (IFT) by MED-EL®. An example of EFI results is shown in Fig. 1(c). However, all these measurements have limitations in providing complete information about stimulus spread. Basically, for TIM/EFI/IFT, the transimpedance is calculated from the measured voltage at the non-stimulating electrode in response to a current pulse at certain timing after the pulse starts, but the measured voltage waveform does not depict a steep rising edge and a plateau. These characteristics are different from the current pulse waveform, since the cochlea as a biological tissue is not purely electrically resistive but contains capacitive elements.^10^ In order to extract more information from impedances measured from CIs, some studies have decomposed the impedance into access resistance and polarization impedance from the measured voltage waveforms, which partly analyzes the shape of the slow rise at the beginning of stimulus pulse and the additional gradual increasing during the constant stimulation level (Fig. S8).^12,13^ However, the measured values of access resistance and polarization impedance are dependent on the duration of pulses due to the non-linearity in the voltage waveforms for either access resistance or polarization impedance measurements, and therefore, this decomposition is not really applicable to short (e.g., <5 *µ*s) or long (e.g., >100 *µ*s) pulses.

In this study, we propose a new method for cochlear impedance characterization using electrochemical impedance spectroscopy^14^ (EIS) to measure cochlear transimpedance. This method can provide the actual complex impedances of cochleas over a range of frequencies and therefore are not conditioned by pulse duration effects. In addition, based on the EIS measurements, an equivalent circuit model for the cochlear transimpedance can be found, which can be applied to any types of pulses with different pulse shapes and pulse durations so that modeling of spread-induced voltage signals under any current stimulus becomes possible. Here, we characterize the cochlear transimpedances of three human cadaveric specimens using EIS and develop a universal circuit model that can be parameterized to fit different specimens. We also use a Laplace transform to simulate spread-induced voltage signals under square pulses and verify the circuit model by comparing the simulated results with experimental measurements from the specimens. To the best of our knowledge, this work is the first study using EIS to characterize cochlear transimpedances and demonstrates a novel and universal method to advance CI stimulus design, which is also applicable to other electrical implants.

## II. EXPERIMENTAL

### A. Cadaveric specimen surgery

Fresh-frozen human cadaveric heads were procured from the Anatomy Gifts Registry (USA) for surgical training and research within a longstanding surgical training facility in our institution. Three fresh-frozen human cadaveric heads were implanted with the same Advanced Bionics® HiFocus 1J lateral-wall electrode array. The cochlea was implanted as it is clinically; a mastoidectomy, posterior tympanotomy, and round window approach were performed. Prior to implantation, the cochlea was flushed with 1.0% saline (GIBCO distilled water, sodium chloride S/3160/60, Fisher Scientific, MA) through a 1-mm opening in the lateral semi-circular canal (LSCC) to remove any debris inside the cochlea.^10^ This has similar electrical conductivity to the normal perilymph that is there. After flushing the cochlea and inserting the electrode array through the round window, the 1-mm opening created in the LSCC was closed off with a non-conductive material (Blu Tack). A ground electrode was placed underneath the temporal muscle. The Advanced Bionics® 1J electrode array has 16 electrodes, with electrode 1 being the most apical electrode and electrode 16 the most basal electrode. As fabricated by its commercial provider, the electrode array used in this study only had odd-number electrodes actually connected to pins out to a D-25 connector. In addition, due to the damage of the electrode array after many implantations, electrode 1 (the most apical one) was broken, and we only had seven working electrodes from the electrode array (i.e., electrode Nos. 3, 5, 7, 9, 11, 13, and 15 [Fig. 1(d)].

### B. Electrochemical impedance spectroscopy (EIS) measurements

**FIG. 2.**
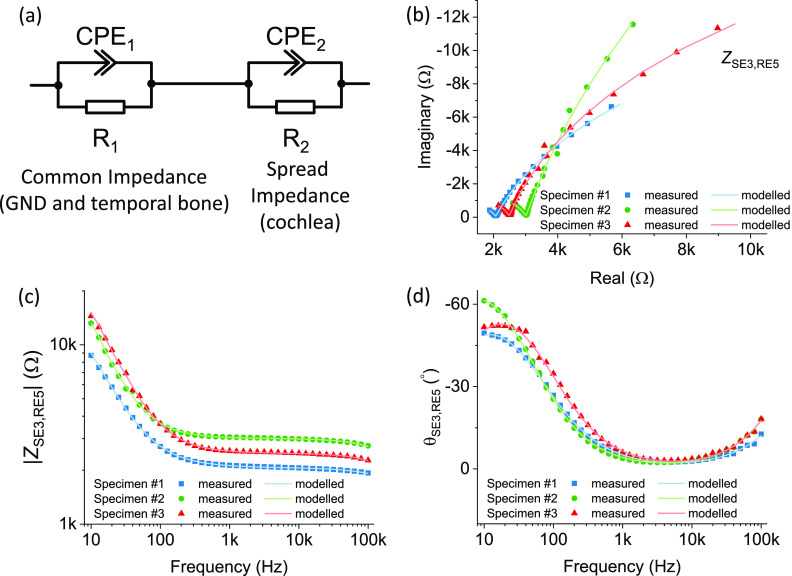
(a) Schematic of the equivalent circuit model for the EIS measurements. (b) The Nyquist plot of EIS measured from three human cadaveric specimens when stimulating from electrode 3 and recording at electrode 5. [(c) and (d)] The Bode plot of EIS measured from three human cadaveric specimens when stimulating from electrode 3 and recording at electrode 5, showing (c) the impedance magnitudes and (d) phases.

After implantation, the 1J electrode array was connected to an impedance analyzer (RS PRO LCR-6100) using a three-terminal configuration with the simulating electrode connected to the high-force terminal, the recording electrode connected to the high-sense terminal, and the ground electrode connected to both the low-force and low-sense terminals. The frequency range was from 10 Hz to 100 kHz, with 10 frequencies per decade (at equivalent logarithmical intervals). An AC signal with the amplitude of 100 mV was used. This frequency range encompasses most of the frequency components of the usual clinical stimulus signals in which the phase durations are typically around 20 *µ*s–200 *µ*s.^15^ In addition, the measurements from 10 Hz to 100 Hz can be used to characterize the low-frequency polarization effect that is commonly seen in voltage waveform recording. The measurement results were analyzed and fitted with an equivalent circuit model, a parallel circuit of a resistor (*R*_1_), and a constant phase element (*CPE*_1_) in series with another parallel circuit of a resistor (*R*_2_) and a constant phase element (*CPE*_2_), as shown in Fig. 2(a). All the data were fitted by ZView® (Scribner Associated Inc.). The measurements were performed at the odd-number electrodes, since the 1J electrode array provided by the manufacturer (Advanced Bionics®) only had these connections from these electrodes to the connector block.

### C. Spread-induced voltage measurements

The 1J electrode array was connected to an CI emulator in which stimuli can be generated and controlled by Bionic Ear Data Collection System (BEDCS) research software. Both the emulator and research software were sourced from Advanced Bionics®. The current stimuli used were charge-balanced biphasic cathodic-leading square pulses, with a duration of 32 *µ*s for each phase and an amplitude of 800 *µ*A. Spread-induced voltage signals were recorded with an oscilloscope (Teledyne LeCroy HDO4054A-MS). The sampling rate was 1 GHz. The results were transmitted to a LabView program and conditioned with a digital Butterworth low-pass filter with a cut-off frequency of 6.25 MHz to remove radio-frequency noises from the CI.

### D. Spread-induced voltage modeling

A Laplace transform was performed on the EIS equivalent circuit model and the biphasic current stimulus, and their product gave the Laplace transform of the spread-induced voltage signal. Then, an inverse Laplace transform was performed to obtain the transient form of the spread-induced voltage signal. We performed the modeling on MATLAB (version R2018b). The modeled results were compared to the experimental measurements.

## III. RESULTS AND DISCUSSION

The EIS results of three human cadaveric cochleas in full heads are depicted in Fig. 2. To avoid confusion from presenting all measurements in one graph, we show the EIS results of three different specimens measured at the same stimulating and recording electrodes (No. 3 and No. 5, respectively), which represented a typical stimulating/recording pair. Comparing the impedances of the three specimens, they are in the same order of magnitude, but specimen No. 1 shows lower overall impedances, as shown in Fig. 2(c). The variations of impedances among different specimens can result from bone compositions, cadaver age, anatomical geometries, CI insertion depth, etc.^16,17^ These factors could not be easily separately investigated. However, impedances of tissues are in the same order of magnitude. The lower impedances of specimen No. 1 than the other two could be attributed to the possible high ion concentrates in bones and/or a shorter electrical pathway from cochlea to the ground electrode. As shown in Fig. 2(b), the Nyquist plot of each specimen dataset demonstrates one pole, where the imaginary component becomes insignificant when compared to the real component. This indicates that the Nyquist plots are likely to contain two semicircles and that the EIS data can be fitted by an impedance circuit model with two resistor–capacitor (RC) circuits in series.^14^ Since objects under test were human specimens and not electrical elements, a capacitor is likely an imperfect representation and we used constant phase elements (CPEs) instead of capacitors, as shown in Fig. 2(a). The mathematical model for the complex impedance of a CPE can be expressed as^14^

$${Ż}_{CPE}\left(\omega \right)=\frac{1}{Y{\left(j\omega \right)}^{p}},$$

where *Y* is the admittance magnitude at 1 rad/s and *p* is the parameter for the constant phase (*θ* = −90*p*°). The equivalent circuit fitted well to the measured data of the three specimens in the Nyquist plots in Fig. 2(b) and the Bode plots in Figs. 2(c) and 2(d). This good agreement suggested that the equivalent circuit might be applicable to different specimens by using parametric variations in the components of the equivalent circuit. In the Bode plots, the cochlear transimpedances demonstrated relatively flat curves in magnitude and close-to-zero results in phase between ∼1 kHz and ∼10 kHz, which corresponded to the zero-imaginary results showed in the Nyquist plots. This indicates that the cochlear transimpedances are dominated by different R-CPE circuits at low frequencies (<1 kHz) and high frequencies (>10 kHz).

**FIG. 3.**
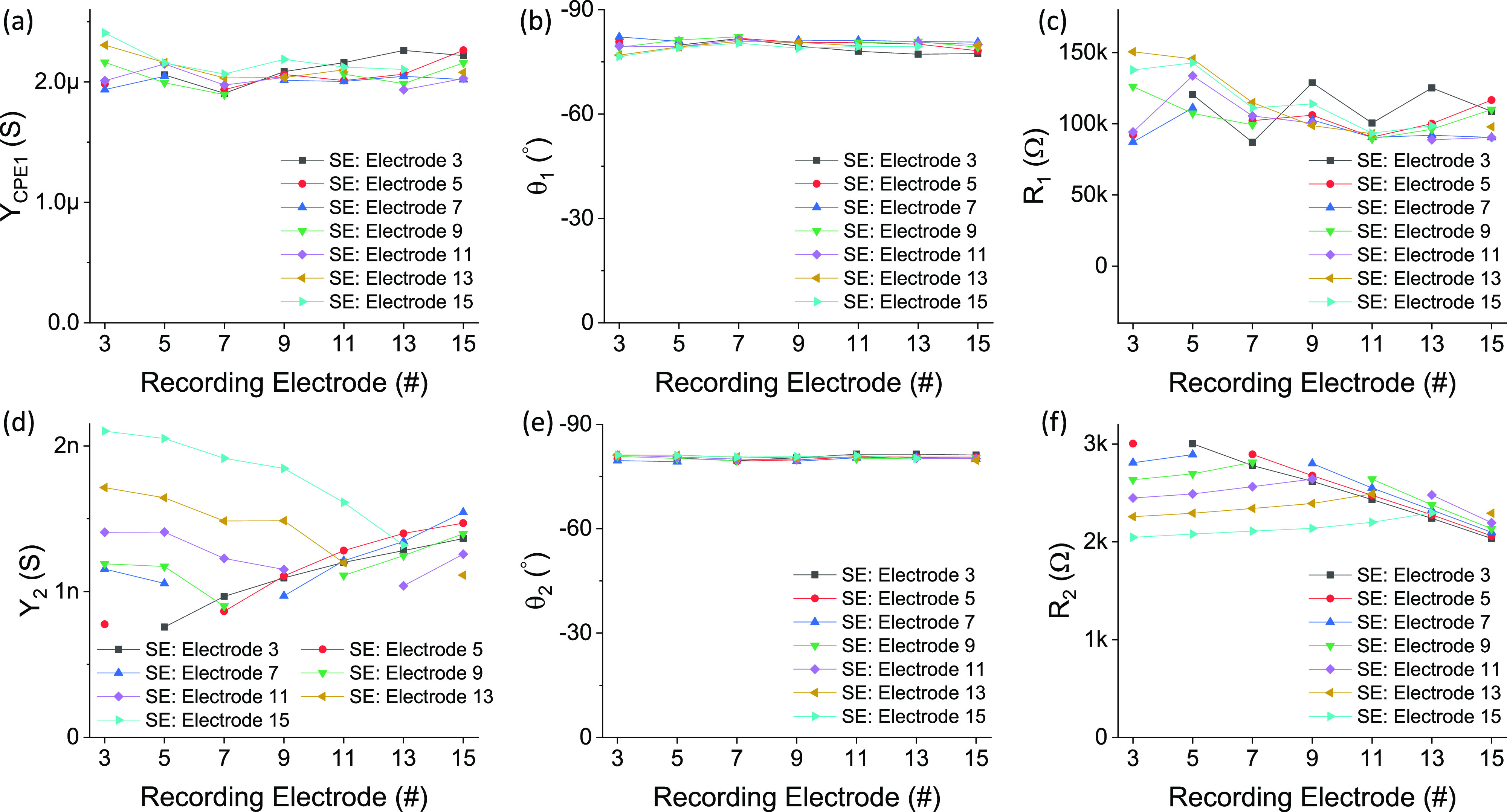
The extracted values for all the components in the equivalent circuit model fitted to the measured EIS in specimen No. 2: (a) the admittance magnitude at 1 rad/s of *CPE*_1_ (*Y*_1_), (b) the constant phase of *CPE*_1_ (*θ*_1_), (c) *R*_1_, (d) the admittance magnitude at 1 rad/s of *CPE*_2_ (*Y*_2_), (e) the constant phase of *CPE*_2_ (*θ*_2_), and (f) *R*_2_.

We further investigated the fittings of the equivalent circuit and found the origins of these two R-CPE circuits. We extracted the parameters for the resistors and CPEs in the measured EIS results of one specimen from all the measurement pairs, as shown in Fig. 3. From the extracted parameters, we observed quite different behaviors between two R-CPE circuits. The first R-CPE circuit (*R*_1_ and *CPE*_1_) seemed constant in all the measured EIS results, i.e., regardless of the locations of the stimulating and recording electrodes, as shown in Figs. 3(a)–3(c). In this case, *R*_1_ and *CPE*_1_ were spread-independent. In contrast, the second R-CPE circuit (*R*_2_ and *CPE*_2_) was more spread-dependent. Obviously, stronger stimulus spread should be seen at places closer to the stimulating electrode and therefore result in higher impedances in the EIS measurements. As shown in Fig. 3(f), *R*_2_ demonstrated hill-like curves with the virtual peaks at each stimulating electrode. Inversely, for *CPE*_2_, the admittance magnitude at 1 rad/s (*Y*_2_) demonstrated valley-like curves with the virtual dips at each stimulating electrode. Interestingly, the constant phases of *CPE*_2_ (*θ*_2_) for different measurement pairs were also independent of the locations of stimulating and recording electrodes. This could be explained by the properties and compositions of a cochlea for ionic conduction being similar along a given cochlear duct. Therefore, although different locations result in the degree of spread being different, the phase delay of spread-induced longitudinal impedances as a result of ion movement in the 1% NaCl solution is not affected. The current pathway for cochlear transimpedance measurements is from cochleas via temporal bones to the remote ground electrode under the temporal muscle. This results in effectively two types of impedances, a common and similar pathway for current returning from each electrode (i.e., from the ground electrode to temporal bones), which is recording electrode independent, and a stimulating/recording-location-dependent pathway (i.e., within cochleas). Therefore, we could suggest that the common pathway corresponds to *R*_1_ and *CPE*_1_, and what varies the transimpedances is the inner cochlear structures that correspond to *R*_2_ and *CPE*_2_. We simulated the impedances of the inner cochlear structures from 10 Hz to 100 kHz and found that the impedances were nearly resistive from 10 Hz to 10 kHz (Fig. S7), which was consistent with the previous report by Spelman *et al.*^18^

We further validated this equivalent circuit and the origins of two R-CPE circuits by extracting the parameters of the equivalent circuit for the EIS measurements in the other two specimens. As shown in Figs. S1 and S2, the parameters of the first R-CPE circuit were also independent of the stimulating and recording electrode locations, as well as the phase of *CPE*_2_, whereas *R*_2_ and *Y*_2_ extracted from specimen Nos. 2 and 3 demonstrated spread-related profiles that were similar to specimen No. 1. More importantly, the extracted parameters of the equivalent circuit were in the same range among all these three specimens. The parameters of *R*_2_ and *CPE*_2_ were different among different specimens due to the differences of intracochlear geometries and slight variations in implantation depth in the specimens. We did observe some differences in the parameters of *R*_1_ and *CPE*_1_ among different specimens (Fig. S3), which could potentially be attributed to the differences of specimens, including the contacts between the ground electrode and cranium, conductivities of skulls and temporal bones, sizes of the head, differing soft tissue pathways, such as muscle bulk,^16,19^ and variations in the day-to-day temperatures during testing of specimens. The extracted parameters in the equivalent circuit can reveal some differences not easily noticeable in Bode plots. As shown in Fig. 3(c), specimen Nos. 2 and 3 demonstrated similar impedances at low frequencies (10 Hz–100 Hz), which were different from that of specimen No. 1. However, when comparing the extracted circuit parameters as shown in Fig. S3, we only found similarity in *Y*_1_, but differences in *θ*_1_ and *R*_1_. In particular, for *R*_1_, specimen No. 3 demonstrated a closer value to specimen No. 1 than specimen No. 2. As previously mentioned, the independent effects of the factors of impedance variations on these circuit parameters are difficult to understand. However, it could be the reason that these factors, though causing differences in *Y*_1_, *θ*_1_, and *R*_1_, could work together and show a combined result, which has similarity in the overall impedance results of specimens Nos. 2 and 3 from 10 Hz to 100 Hz. Despite the differences, the parameters of *R*_1_ and *CPE*_1_ among different specimens were in the same ranges of magnitudes (Fig. S3).

With this well-fitted equivalent circuit model to human cochlear transimpedances, we simulated spread-induced voltage signals. This was the primary purpose of the characterizations of human cochlear transimpedances, i.e., to understand the stimulus spread, including the amplitude and shape of signals. According to its definition in electronics, transimpedance is the ratio of the resultant voltage at the output terminal to the current at the input terminal.^20^ Since we have information for cochlear transimpedances, we have the relation between spread-induced voltage and stimulating current at all stimulating/recording pairs as^14^ ${\dot{V}}_{k}\left(s\right)={İ}_{j}\left(s\right){Ż}_{j,k}\left(s\right)$, where ${İ}_{j}\left(s\right)$ and ${\dot{V}}_{k}\left(s\right)$ are the stimulating current and spread-induced voltage at the *j*^th^ and *k*^th^ electrode of a CI, respectively, and ${Ż}_{j,k}\left(s\right)$ is the transimpedance when stimulating at the *j*th electrode and recording at the *k*th electrode (*j* ≠ *k*), *s* is the complex variable. Note that all these terms are in the *s* domain. For the stimulus, we considered a symmetric cathodic-leading biphasic square pulse, which is commonly used clinically. The Laplace transform of the biphasic current stimulus from the *i*th electrode can be expressed as^21^ ${İ}_{j}\left(s\right)=-\frac{i_{j,amp}}{s}\left(1-2e^{-sT_{0}}+e^{-2sT_{0}}\right)$, where *i*_j,amp_ is the current amplitude and *T*_0_ is the duration of each phase. As for the transimpedances, *Z*_j,k_(*s*) can be expressed as ${Ż}_{j,k}\left(s\right)={Ż}_{CPE1,j,k}\left(s\right)+\frac{{Ż}_{CPE2,j,k}\left(s\right)\cdot R_{2,j,k}}{{Ż}_{CPE2,j,k}\left(s\right)\;+\;R_{2,j,k}}$, where the subscripts *j*, *k* denote locations of the stimulating (*j*th) and recording (*k*th) electrodes for the parameters (*j* ≠ *k*). The spread-induced voltage in the *s* domain is simply the production of current stimulus and transimpedance in the *s* domain. To obtain the spread-induced voltage in the time domain, we performed an inverse Laplace transform, $v_{k}\left(t\right)=L^{-1}\left\{{İ}_{j}\left(s\right){Ż}_{j,k}\left(s\right)\right\}$, and found

$$\begin{array}{ll} v_{k}\left(t\right) & =-i_{j,amp}\left\{\frac{t^{p_{1,j,k}}}{Y_{1,j,k}\Gamma \left(p_{1,j,k}+1\right)}+\frac{t^{p_{2,j,k}}}{Y_{2,j,k}}E_{p_{2,j,k},p_{2,j,k}+1}\left(-\frac{t^{p_{2,j,k}}}{Y_{2,j,k}R_{2,j,k}}\right)\right\}\\ & \;*\left\{\delta \left(t\right)-2\delta \left(t-T_{0}\right)+\delta \left(t-2T_{0}\right)\right\}, \end{array}$$

where $\Gamma \left(x\right)=\int_{0}^{+\infty }t^{x-1}e^{-t}dt$ is the Gamma function and $E_{\alpha ,\beta }\left(x\right)=\sum_{l=0}^{\infty }\frac{x^{l}}{\Gamma \left(\alpha l+\beta \right)}$ is the Mittag–Leffler function. All the detailed mathematical derivations can be found in the supplementary material [Eqs. (S1.1)–(S1.16)].

**FIG. 4.**
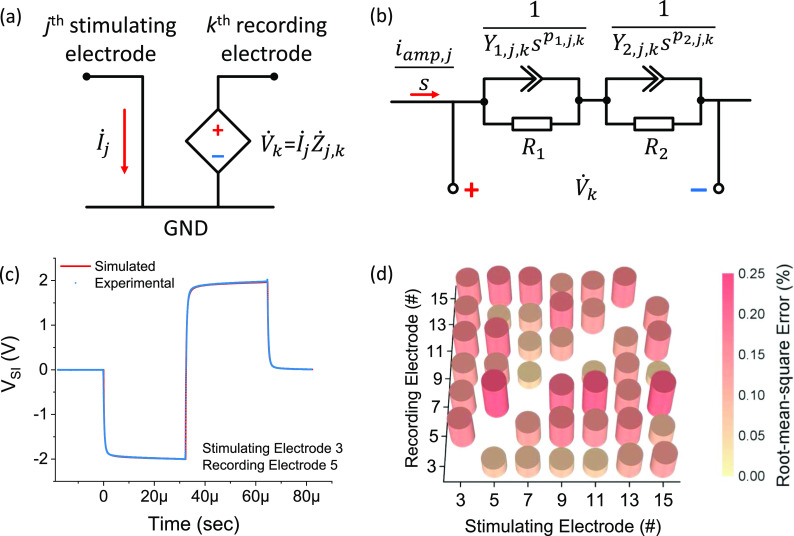
(a) The relation of current stimulus (${İ}_{j}$), voltage response (${\dot{V}}_{k}$), and transimpedance (${Ż}_{j,k}$) in the frequency domain. (b) The Laplace transforms of current stimulus, voltage response, and the equivalent circuit for cochlear transimpedance. (c) The simulated (red curve) and measured (blue dot) spread-induced voltage response (*V*_SI_) as a function of time, showing the good agreement between the simulated and experimental results. (d) The relative errors between the simulated and experimental results for all the simulating and recording pairs.

A typical simulated spread-induced voltage signal is shown in Fig. 4(c) and was compared with experimental data measured from the same specimen. The simulated data and the experimental data were almost completely overlap as plotted in Fig. 4(c), with a root-mean-square (RMS) error of 0.3 mV, i.e., a RMS relative error of 0.08%. We also calculated the RMS errors for all the measurement pairs and found that the errors were below 0.25%. For all the specimens, the average RMS errors were 0.13 ± 0.06%, 0.33 ± 0.14%, and 0.36 ± 0.14%, with a maximum of 0.60% (Figs. S4–S6). The agreement between the simulated and experimental data suggested that the equivalent circuit with two R-CPE circuits might be a good presentation of human cochlear transimpedances, not only for EIS characterizations but also for transient spread-induced voltage measurements. More importantly, since this equivalent circuit can represent cochlear transimpedances at all frequencies, it can be used to simulate the spread-induced voltage signals from stimuli of any pulse shape, duration, and amplitude.

**FIG. 5.**
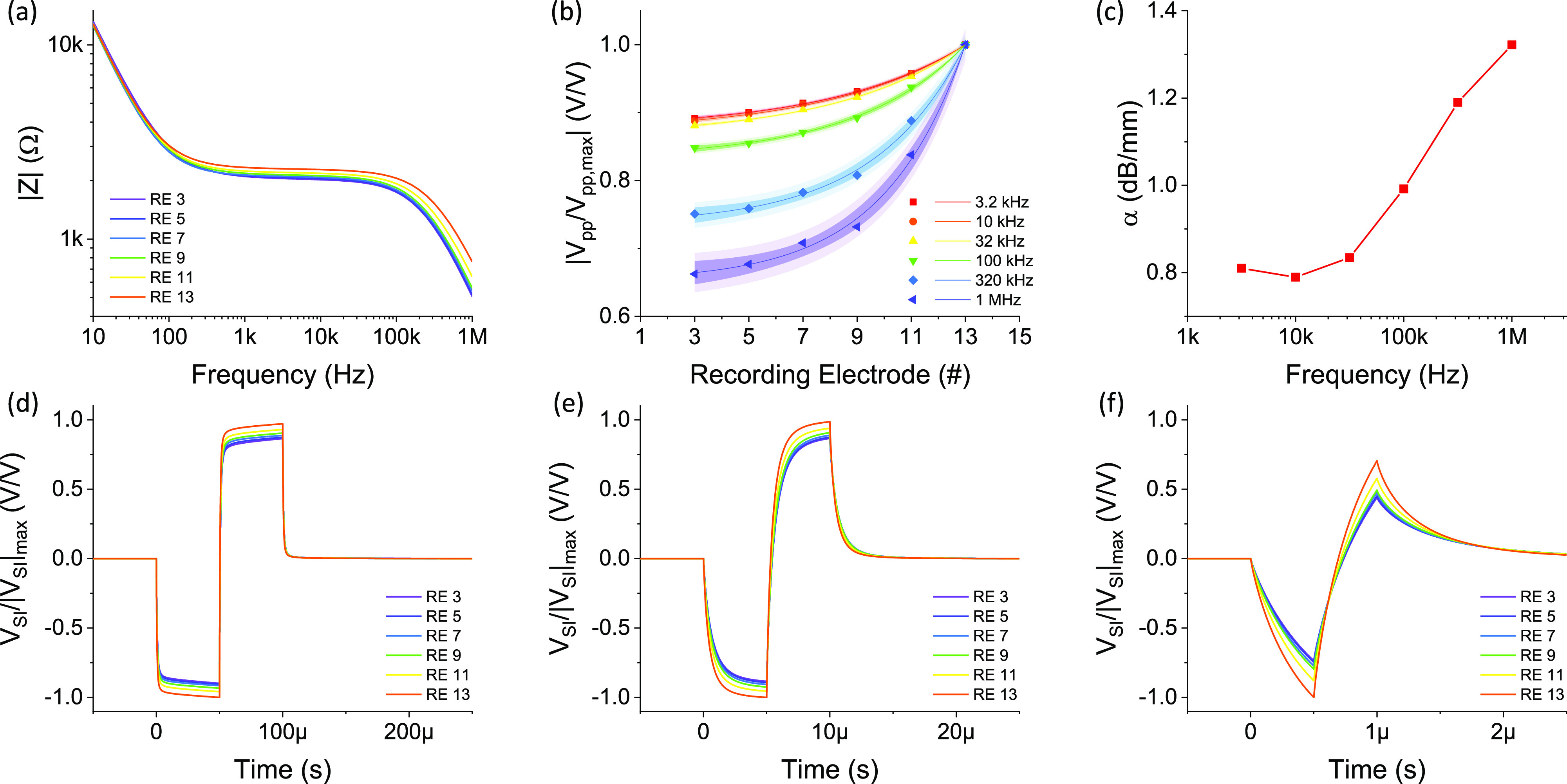
(a) Simulated transimpedance magnitude spectra when stimulating electrode 15. (b) Normalized spread-induced voltage amplitude under stimulations with different frequencies. (c) Stimulus focusing ability under sine-wave stimulations with different frequencies. [(d)–(f)] The transient normalized spread-induced voltage responses under biphasic pulse stimuli with different pulse durations, (d) 100 *µ*s, (e) 10 *µ*s, and (f) 1 *µ*s.

Here, we show an example of simulation and how spread is affected by different pulse durations. Basically, the narrower the pulse durations are, the higher the frequencies of the signals that lie in the spectrum. The cochlear transimpedance spectra under stimulation at electrode 15 are depicted in Fig. 5(a). The cochlear transimpedances at the low frequencies (<100 Hz) were quite similar among the measurements at different recording locations, which indicated a similar level of voltage response around the entire cochlea and a significant electrical spread. At the middle-range frequencies (1 kHz–32 kHz), the cochlear transimpedances were lower at the recording locations farther away from the stimulating electrode, which suggested that spread could be partly reduced in this frequency range, but the spectrum at each stimulating/recording pair was relatively constant across these frequencies. At the high frequencies (>32 kHz), it is noteworthy that the curves were fanned out toward higher frequencies, i.e., the cochlear transimpedances dropped faster at the recording locations farther away from the stimulating electrode. This was probably because at the farther-away locations, the AC signal delays could be longer, and the transimpedances contained more capacitive elements that caused the transimpedance magnitudes to drop faster. From this, the electrical stimulus can be more focused if the stimulus is a higher-frequency signal, which was confirmed by the plots to visualize this spread in the normalized spread-induced voltage response distributions using pulses with different frequencies in Fig. 5(b). As shown in Fig. 5(c), the spatial spread was similar at frequencies below 32 kHz and started to decrease over 32 kHz. We simulated the normalized transient spread-induced voltage responses under symmetric biphasic stimuli with different pulse durations (i.e., 100 *µ*s, 10 *µ*s, and 1 *µ*s), as shown in Figs. 5(d)–5(f). It can be seen that the amplitudes of voltage responses at the recording locations farther away from the stimulating electrode were reduced in the case of 1-*µ*s pulses compared to 100-*µ*s pulses. It is also noteworthy that the voltage response became more asymmetric under pulses with shorter pulse durations. This may be useful for auditory nerve stimulation, where the central nerves and peripheral nerves respond to different polarities of stimuli,^22–24^ while the feasibility of delivering high-frequency pulses and the actual nerve responses need to be further tested.

## IV. CONCLUSION

In conclusion, we have characterized complex impedance spectra of human cadaveric cochleas using a cochlear implant and found an equivalent circuit model for the measured impedances. The circuit model indicated two separate parts of impedances of which one is spread-induced and the other is spread-independent. The circuit model could be used for modeling electrical stimulus spread signals, which were verified with stimulations and recordings from a cochlear implant. Therefore, with the cochlear impedance spectroscopy measurements and the equivalent circuit, one could design new cochlear implant stimuli and predict the voltage response and spread distribution. Based on this, stimuli can be customized for different cochlear implant users, and more types of stimuli can be tested before applying to them *in vivo*. As shown in this study, using an ultra-short phase duration of 0.5 *µ*s can significantly suppress the stimulus spread, which provides an example for cochlear implant clinics to reduce spectral blurring and improve cochlear implant performance. In addition, the spread-independent component could be clinically useful to diagnose problems with the ground electrodes. Moreover, this method is also applicable to other medical implants using electrical stimuli.

When coupled to the existing electrical characterization tools integrated with cochlear implants, this technology could reveal more information about cochleas for cochlear implant users, with potential for cochlear implant troubleshooting, such as distinguishing problems with intracochlear electrodes or extracochlear ground electrode and identifying the conditions (such as fibrosis or soft tissue ingrowth) surrounding the electrodes. For future work, we will test the universality of this method and model for cochlear implants with different designs from different manufacturers, and further explore the stimulus design to suppress electrical spread and enhance stimulation efficiency, and correlate the results with psychoacoustic measurements in patients with CIs.

## SUPPLEMENTARY MATERIAL

See the supplementary material for the complete mathematical derivations and impedance results of other specimens that are not shown.

## DATA AVAILABILITY

The data that support the findings of this study are available within the article and its supplementary material.
